# Editorial: Osteoporosis and the Role of Muscle

**DOI:** 10.3389/fendo.2022.951298

**Published:** 2022-06-27

**Authors:** Marco Brotto, Marco Invernizzi, Alex Ireland, Gordon L. Klein

**Affiliations:** ^1^ Director of Bone-Muscle Research Center, University of Texas at Arlington, Arlington, TX, United States; ^2^ Department of Health Sciences, University of Eastern Piedmont, Novara, Italy; ^3^ Senior Lecturer, Manchester Metropolitan University, Manchester, United Kingdom; ^4^ Senior Scientist and Adjunct Professor, Department of Orthopaedic Surgery and Rehabilitation, University of Texas Medical Branch, Galveston, TX, United States

**Keywords:** osteoporosis, muscle, biomechanics, endocrinology, molecular biology, Cell Biology, nutrition, Pharmacology

This Research Topic, Osteoporosis and the Role of Muscle, began as an exploration of the effect bone resorption had on muscle wasting and the contribution of the latter to falls and fragility fractures. As contributions began arriving for this topic, it became clear that the subject had many more aspects than was initially conceived. In fact, papers have approached this topic from the standpoints of creation of a new model to study bone and muscle crosstalk, neurologic illnesses and spinal cord injury as a means to understanding the relationships between bone and muscle, genetics, cell biology, biomechanics and nutrition, along with the original translational concept of the biochemical interactions of bone and muscle.

Biguetti et al. developed a mouse model involving surgical injury to the vastus lateralis muscle and the femur in which to study simultaneous healing of muscle and bone in relation to age, sex, and the 5-lipoxygenase pathway, knockout of which demonstrated less inflammation than in wild type animals.

Lin et al. using a weighted gene co-expression analysis of micro RNAs, messenger RNAs and genes that are osteoporosis related to identify a potential micro- and messenger RNA regulatory network. While this study did not specifically mention muscle, if these same bioinformatics techniques were to be used to develop a micro and messenger RNA regulatory network of gene expression in muscle, it might be possible to draw an intriguing comparison of regulatory networks for gene expression in bone and muscle and their relative actions in osteoporosis, as well as other related conditions.

In a slightly different approach to muscle-bone interaction studies, Zhang et al. examined the effect of vitamin D supplementation on hand grip strength in a review and meta-analysis of 13 randomized controlled studies in post-menopausal women. The outcome demonstrated an improvement in handgrip strength, though not in timed Up and Go studies, raising the possibility that vitamin D supplementation may selectively improve only certain muscle function in the elderly.

Studies in biomechanical influences of muscle on bone were reported by Luscher et al. in trained long-distance runners compared to untrained controls using serial peripheral quantitative computed tomography. While the runners had greater bone strength and stiffness in the tibia, they also had lower lateral bending stiffness in the fibula. The authors suggest that this adaptation could improve energy storage during locomotion, implying a novel biomechanical role of the skeleton beyond transmitting force and resisting fracture. Nutrition was also examined in high-performing athletes as Heikura et al. demonstrated that a ketogenic diet in elite race walkers impairs biomarkers of bone formation while increasing markers of bone resorption, potentially leading to a low-formation, high-resorption state following exercise, making the case for further study of the biochemical effects of a high fat, low carbohydrate diet on the musculoskeletal system of elite athletes.

Two articles examining cell biologic aspects of muscle and bone metabolism included one by Xu et al. that finds that hypoxia-inducible factor-1 alpha (HIF-1α) in MC3T3 osteoblast-like cells exposed to dexamethasone triggers a pathway *via* pyruvate dehydrogenase kinase-1 (PDK-1) that will phosphorylate the AKT/mTOR anabolic pathway. This pathway is suppressed by dexamethasone exposure. It would not be surprising if this same pathway is active in skeletal muscle as well as glucocorticoid exposure is known to produce oxidative stress in both bone and muscle and result in up-regulation of the FOXO genes that that impair osteoblastogenesis and muscle protein synthesis (1, 2).

A review by Chen et al. examines the role of N^6^ methyladenosine (m^6^A) in the form of m^6^A methyltransferase METTL_3_, which is involved in the osteogenic versus adipogenic fate of marrow stem cells. Knockdown of METTL_3_ suppresses expression of runx2 and osterix, which are critical steps in osteoblastogenesis and can also reduce expression of VEGF. M^6^A in muscle myoblasts controls their transition to other muscle cell states (3, 4). This is an epigenetic factor that exists in both bone and skeletal muscle and the coordination of actions in both tissues is worth further study.

In manuscripts covering the spinal cord and central nervous system, Iolascon et al. discuss the current treatments available for neuromuscular disease and makes us appreciate the complexity of interactions among muscle, bone, and nerves. It is clear that we have much to learn before we can propose a truly integrated model of muscle, bone and nerve, in the regulation of muscle and bone mass and function. Invernizzi et al. in the meantime review the available treatments for the joint loss of bone and muscle in spinal cord injury. While this review is state of the art, it also leaves us with the need to investigate more thoroughly the relationship between muscle and bone loss in neurologic conditions.

Finally, we return to the original idea in the role of muscle in osteoporosis. That is, that the bone on resorption liberates transforming growth factor beta (TGF-β), as shown by Pin et al. in pediatric burns and by Essex et al. in cisplatin-treated mice. The use of anti-resorptives, pamidronate in the case of pediatric burns and zoledronic acid in the case of cancer-treated mice, protected the bone and muscle mass in each case, demonstrating that is a factor released by bone that contributes to, if not being wholly responsible for, muscle wasting in these conditions. In the case of myoblast studies of Pin et al. the TGF-β released from bone suppressed the phosphorylation of the anabolic AKT/mTor pathway and increased expression of ubiquitin of the catabolic ubiquitin ligase pathway. These findings explain the original report of the characterization of muscle protein preservation with use of bisphosphonates in pediatric burns (5)

This entire collection of articles provides for the first time a focus on a variety of different aspects of muscle and bone interaction in a variety of conditions and with more study of this emerging area we hope that more definitive treatments can be identified that can alter the mechanisms that lead to bone loss and consequent muscle wasting.

## Author Contributions

All authors listed have made a substantial, direct, and intellectual contribution to the work and approved it for publication.

## Conflict of Interest

The authors declare that the research was conducted in the absence of any commercial or financial relationships that could be construed as a potential conflict of interest.

## Publisher’s Note

All claims expressed in this article are solely those of the authors and do not necessarily represent those of their affiliated organizations, or those of the publisher, the editors and the reviewers. Any product that may be evaluated in this article, or claim that may be made by its manufacturer, is not guaranteed or endorsed by the publisher.
